# Predictive value of stathmin-1 and osteopontin expression for taxan resistance in metastatic castrate-resistant prostate cancer

**DOI:** 10.12669/pjms.333.12559

**Published:** 2017

**Authors:** Asude Aksoy, Gokhan Artas, Omur Gokmen Sevindik

**Affiliations:** 1 Asude Aksoy, Department of Medical Oncology, Medical Faculty, Firat University, Elazig, Turkey; 2 Gokhan Artas, Department of Pathology, Medical Faculty, Firat University, Elazig, Turkey; 3 Omur Gokmen Sevindik, Department of Hematology, Medical Faculty, Firat University, Elazig, Turkey

**Keywords:** Osteopontin, Stathmin-1, Taxane resistance, Metastatic castrate-resistant prostate cancer, Survival

## Abstract

**Objective:**

Several pathways are known to be activated during metastasis and treatment of cancer. We investigated the role of osteopontin (OPN) and stathmin-1 (STHMN1) in metastatic castrate-resistant (mCRPC).

**Methods:**

We included 30 patients who received at least 6 cycles of taxane regimen for metastatic mPC in the present study. For this study retrospective data was taken from Firat University, Faculty of Medicine, Medical Oncology Department between 2009 and 2015. OPN expression and STHMN1 expression were retrospectively evaluated by immunohistochemical staining in biopsy specimens. The relationship between the expression levels of OPN and STMN1 and the response to taxane based regimen and survival was analyzed.

**Results:**

There was mild or strong overexpression of OPN and STHMN1 in all the patients. STHMN1 expression was mildly positive (+2) in four of the cases (13.2%) while it was strongly positive (+3) in 25 (83.4%) cases. Similarly, OPN expression was mildly positive (+2) and strongly positive (+3) in five (16.6%) and 25 (87.4%) patients, respectively. There was no significant correlation between the expression levels of STHMN1 and OPN, survival, and response to taxane based regimen (p>0.05); however, OPN overexpression showed a significant correlation with lower Gleason scores (GS) (p:0.032).

**Conclusions:**

STHMN1 and OPN may be prognostic markers although they are not predictive markers of response to treatment in mCRPC. The overexpression of OPN may help identifying patients with lower GS.

## INTRODUCTION

Prostate cancer is the second most common cancer and the leading cause of cancer-related death in men worldwide. Continuous androgen deprivation therapy (ADT) is recommended as first-line treatment for metastatic and hormone-naive disease. These patients eventually gain resistance to ADT. The level of PSA of patients increase, the levels of testosterone areat castration, and the disease progress in a few years. Currently, chemotherapy (CT) is often recommended in combination with ADT as the initial treatment for metastatic prostate cancer castre-resistance prostate cancer (mCRPC) with high tumor volume.^1^

Taxane based regimen (TBR) is the first option in patients with metastatic prostate cancer. Taxanes inhibit mitosis by decreasing the depolymerization of β-tubulin.^1^,^2^ There are several studies showing improved overall survival (OS) with TBR while some patients may not respond to this treatment.

Some signaling pathways such as phosphoinositide 3-kinase (PI3K) and hedgehog are activated during the metastatic process and resistance to drugs in various cancers. OPN as well as STMHN1 have been reported to be playing an important role through activation of the PI3K/Akt pathway in metastasis and resistance to chemotherapeutics (CTs) in cell lines.^3^-^6^

There are several studies on the effects of vinca alkaloids on microtubules in cell lines, and on clinical outcomes with tailored treatment modification according to predictive markers in several types of cancer.^3^,^7^ Overexpression of STHMN1 and/or OPN may be a marker of proliferation and resistance to TBR in mCRPC.

We retrospectively described the roles of STHMN1 and OPN expression in the metastatic process and in predicting the response to TBR. This relationship has not been reported in the literature to date.

## METHODS

All the cases were provided retrospectively from the records of Firat University, Faculty of Medicine, Medical Oncology Department between 2009 and 2015. This study was approved by the Ethical Committee of Firat University. The patients included in the present study were diagnosed with PC and had received at least 6 cycles of TBR (75 mg, taxane mg/m^2^, every 21 days, intravenous infusion, together with 10 mg/day prednisone, peroral, continue) for mCRPC. All cases were evaluated every three cycles.

A total of 30 cases were included in the study. The patients were divided into two groups as responders and non-responders. The group of responders was evaluated in three categories as patients with response, stable disease and flare phenomenon based on the PSA working group consensus criteria as follows: a) Response: ≥50% PSA reduction from baseline, b) Stable disease: <50-0% PSA reduction from baseline or absence of any reduction, c) Flare phenomenon: ≥50% PSA reduction from baseline followed by increased PSA levels with subsequent PSA reduction below the baseline values.^8^ In addition, patients were also stratified into two groups according to GS as the intermediate-risk group (GS: 7) and the as high-risk group (GS: 8-10).^1^,^9^

Immunohistochemical (IHC) evaluation: 4-5 μm sections of paraffin blocks were obtained from the cases. The sections were placed on slides coated with poly-L-lysine. Slides were incubated for 10-15 minutes at 60°C in the incubator. Preparations were stained with OPN (Boster, rabbit polyclonal anti-spp1, 100 µL, 1/20, USA) and Bcl-2 (Boster, rabbit Ig G polyclonal antibody for stathmin (STHMN1), 100 µL, 1/50, USA) by means of an automated staining device (ventana medical system, SN: 712299, REF: 750-700, Arizona, USA). Positive controls were prepared with endometrial cancer tissues for STHMN1 and OPN antibodies, and we also used normal prostate epithelium as internal positive control to both antibodies. The slides were evaluated by an independent, blinded pathologist to re-confirm the diagnosis, GS, and the outcome of patients. Slides stained for STHMN1 and OPN were evaluated under a light microscope. STHMN1 and OPN expression ratios were examined in 4 categories:It was identified as 0 (no staining) negative; <10%, “+” weak; 10-50%, “++” mild; and >50%, “+++” extensive). The staining intensity was assessed and scored as negative (no staining), weak(+1, +2) and strong (+3).^10^

The software package, International Business Machines, Statistical Package for the Social Sciences, 22.0, was used for the statistical analysis of the data. OS was defined as the time from the date of diagnosis to death or the final visit. Kaplan-Meier analysis was used to assess survival and the log-rank test was usedfor statistical analysis. Values of p<0.05 with a 95% confidence interval were considered as statistically significant.

## RESULTS

**Fig.1a:**
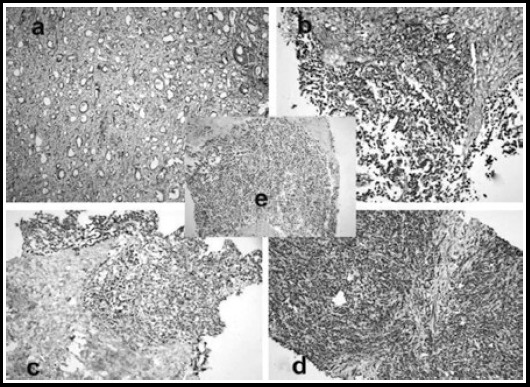
Osteopontin, SS: 2 (Gleason Score 3+4 (Haematoxylin and Eosin (HE) X 100), Immunoperoxidase X 100) 1b: Osteopontin SS:3, (Gleason Score 5+5 (HE X 200), Immunoperoxidase X 200), 1c. Stathmin-1, SS:2, (Gleason Score 5+5 (HE X 200), Immunoperoxidase X 200), 1d: Stathmin-1, SS:3, (Gleason Score 5+5 (HE X 200), Immunoperoxidase X 200), 1e: The speciemens of prostate transurethral resection, aciner adenocarcinoma (Gleason Score 5+5 (HE X200), Immunuperoxidase X 200)

OPN and STMN1 expressions are shown in (Fig.1). OPN and STHMN1 were observed to be expressed in all of the samples. Eighteen (60%) of the patients were in the non-responder groups to both of OPN and STHMN1.

**Table-I:** The relationship between OPN, GS, and response to taxan based regimen.

*Variables*	*n, (%)*	*SS of OPN*		*p values*

		*Mildly n,(%)*	*Strongly n,(%)*	
Responder status			0.622	
Responders	12(40)	1 (8.3)	11 (91.7)	
Non responders	18(60)	4 (22.2)	14 (77.8)	
Gleason Score (GS)#				0.032
GS-Intermediate Risk	13 (43.3)	0	13 (100)	
GS-High Risk	17(56.7)	5 (29.4)	12 (70.6)	

***Abbreviations***: SS: Staining Score, GS: Gleason Score, OPN: Osteopontin, GS-Intermediate Risk: GS 7, GS-High Risk: GS (8, 9, 10), (*According to AJCC 7)1

# According to Pearson’s chi-square test.

**Table-II:** The relationship between STHMN1, GS, and response to taxan based regimen.

*Variables*	*n, (%)*	*SS of STHMN1*		*p values*

		*Mildly n,(%)*	*Strongly n, (%)*	
Responder status				0.661
Responders	12 (40)	2 (16.7)	10 (83.3)	
Non responders	18 (60)	2 (11.1)	16 (88.9)	
Gleason Score (GS)#				0.113
GS-Intermediate Risk	13 (43.3)	0	13 (100)	
GS-High Risk	17 (56.7)	4 (23.5)	13 (76.5)	

***Abbreviations:*** SS: Staining Score, GS: Gleason Score, STHMN1: Stathmin-1, GS-Intermediate Risk: GS 7, GS-High Risk: GS (8, 9, 10), (*According to AJCC 7)1

# According to Pearson’s chi-square test.

**Fig.2a:**
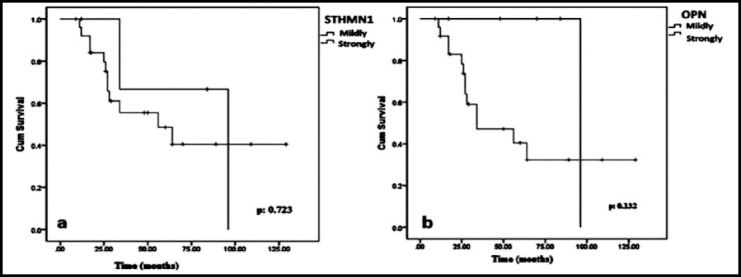
Overall survival graphic (OS) according to stathmin-1 (STHMN1) expression, 2b: Overall survival graphic (OS) according to osteopontin (OPN) expression.

**Fig.3:**
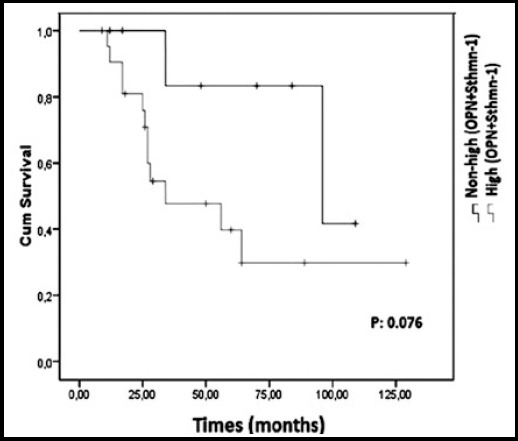
Overall survival graphic (OS) according to overexpressions (stathmin-1 (STHMN1) expression + osteopontin (OPN)) expression and non-high expressions (STHMN-1 + OPN).

The longest survival was 129 months. Median survival was 96 months (95% CI: not estimated) for the mild STHMN1 expression group (EG) and 56 months (95% CI: 11.375-100.625) for the strong STHMN1 EG. In this study, STHMN1 EGs showed no significant correlation with OS, (p-values: 0.723), (Fig.2a). Median survival was 96 months (95% CI: not estimated) for the mild OPN EG and 34 months (95% CI: 0.528-67.483) for the strong OPN EG. There was no significant correlation between the OPN EGs and OS (p-values: 0.132), (Fig.2b). When it was assessed overall survival (OS) according to together expressions of OPN and STHMN1(high OPN and high STHMN1 as one group; high OPN and low STHMN1, low OPN and high STHMN1, low OPN and low STHMN1 as another one group), none of combine group (OPN, STHMN1) predicted OS (p>0.005), (Fig.3).

Multivariate analysis by Cox proportional hazards model revealed that none of the expression levels of OPN or STHMN1 predicted OS (HR: 0.24, 95% CI: 0.018-3.269, p-value: 0.28), (HR: 1.64, 95% CI: 0.24-10.91, p-value: 0.60, respectively).

Median survival was 28 months (95% CI: 18. 49-37.50) in the intermediate-risk GS group and 96 months (95% CI: 49.13-142.86) in the high-risk GS group. There was no statistically significant difference between the GS groups in terms of OS as assessed by the Kaplan-Meier method (p-value: 0.156).

## DISCUSSION

There are limited options for treatment with cytotoxic regimens as prostate cancer is a highly chemoresistant malignancy.^1^ Currently, TBR plays a major role in the treatment of metastatic prostate cancer. Although this cytotoxic agent has been demonstrated to improve OS, it is associated with several difficulties such as dose-dependent adverse reactions, CT resistance and no response to treatment.^1^,^2^,^11^

The underlying molecular mechanisms of CT resistance has not been fully clarified. Genetic and non-genetic alterations have been suspected to be involved in the development of drug resistance in previous studies.^12^,^13^ Although this resistance appears to be multifactorial; defects in tubulin and microtubule-associated proteins (MAPs), dysregulated cell cycle and apoptotic signaling pathways are among the factors thought to be responsible for taxane resistance.^14^ Researchers have demonstrated the relationship between taxane resistance and the overexpression of MAPs, tau protein, STHMN1 and OPN with in vitro and in vivo studies.^4^,^6^ We investigated whether STHMN1 and OPN expressions could be predictive markers for cancer metastasis and resistance to TBR in mCRPC. It has been reported that OPN plays an important role in the progression of several cancers through the regulation of vascular endothelial growth factor (VEGF) release in the PI3K signaling pathway and the effectiveness of various physiological processes.^4^,^15^

Several studies have shown the interaction between OPN and VEGF in the metastatic process and the existing/acquired resistance to CTs.^11^,^12^,^15^ Researchers reported a potential relationship between proliferation, tumorigenicity and level of OPN expression in cell line studies.^4^ In our study, we also found positive OPN expression in all patients tissue samples. Similar to the literature reports, strong OPN expression was identified in 25/30 (83.4%) of the cases. Although an important role has been shown for OPN in regulating chemotherapeutic drug resistance through increased drug transporter expression, no relationship was observed between OPN and response to treatment in our study. Long-term treatment with cytotoxic drugs may further upregulate OPN release from tumor cells. Therefore, researchers suggested that OPN could be a therapeutic target for cancer treatment and avoid drug resistance. In recent studies, OPN knockdown has been shown to increase the activity of CT drugs and inhibit p-glycoprotein expression. The researchers also found that the levels of OPN could be increased, which were antagonized by the addition of various chemical agents in PI3K/AKT pathway, during endogenous secretion of OPN in the prostate cancer cells line.^4^,^16^,^17^ Most of the studies which have demonstrated findings associated with resistance to CTs are mainly cell culture studies rather than definitive clinical studies. Hence, we focused on the OPN expression level as a possible marker of drug resistance. However, our results indicate no relationship between OPN and response to TBR while a statistically significant difference is seen in terms of increased OPN overexpression with low GS in contrast to the findings of some previous studies.^18^

**Table-III:** The relationship between STHMN1 and GS.

Gleason Score (GS)*	*n, (%)*	*SS of STHMN1*		*p values*

		*Mildly n,(%)*	*Strongly n, (%)*	
Group 2 (3+4)= 7	3 (10)	0	3 (100)	0.174
Group 3 (4+3)= 7	9 (30)	0	9 (100)	
Group 4 (4+4),(3+5), (5+3)= 8	5 (16.7)	2 (40)	3 (60)	
Group 5 (5+5),(4+5), (5+4)= 9, 10	13 (43.3)	2 (15.4)	11 (84.6)	

***Abbreviations:*** STHMN1: Stathmin-1, SS: Staining Score,

* GS: According to new classification Gleason Score.^9^

**Table-IV:** The relationship between OPN and GS.

Gleason Score (GS)*	*n, (%)*	*SS of OPN*		*p values*

		*Mildly n,(%)*	*Strongly n,(%)*	
Group 2 (3+4)= 7	3 (10)	0	3 (100)	0.231
Group 3 (4+3)= 7	9 (30)	0	9 (100)	
Group 4 (4+4),(3+5), (5+3)= 8	5 (16.7)	1 (20)	4 (80)	
Group 5 (5+5),(4+5), (5+4)= 9, 10	13 (43.3)	4 (30.8)	9 (69.2)	

***Abbreviations:*** OPN: Osteopontin, SS: Staining Score,

* GS: According to new classification Gleason Score.^9^

**Table-V:** Cox Proportional Multivariate Hazard Models for OS in patients with mCRPC.

	*P*	*HR*	*95 % CI*
Response status	0.28	0.24	0.018-3.269
PSA	0.35	0.57	0.181-1.839
* GS	0.39	0.60	0.190-1.927
OPN	0.28	0.24	0.018-3.269

***Abbreviations:*** OPN: Osteopontin,

* GS: According to new classification Gleason Score.^9^ PSA: Prostate specific antigen, CI: Confidence interval, mCRPC: Metastatic castrate- resistance prostate cancer

**Table-VI:** Cox Proportional Multivariate Hazard Models for OS in patients with mCRPC.

	*P*	*HR*	*95 % CI*
Response status	0.32	0.54	0.165-1.81
PSA	0.60	1.00	0.998-1.00
* GS	0.14	0.39	0.11-1376
STHMN-1	0.60	1.64	0.24-10.91

***Abbreviations***: STHMN1: Stathmin-1,

* GS: According to new classification Gleason Score.^9^ PSA: Prostate specific antigen, CI: Confidence interval, mCRPC: Metastatic castrate- resistance prostate cancer

STHMN1 is a major cytosolic phosphoprotein and a microtubule-depolymerizing molecule involved in the metastatic process of various cancers; however, the mechanism of its regulation has not been fully clarified. The activity of STHMN1 is controlled by PI3K, which requires maintaining a stable microtubule network during migration.^6^ Phosphorylation of STHMN1 is mediated by a number of protein kinases including p27, an inhibitor of cyclin-dependent kinase complexes. In the absence of PI3K/Akt activity, captured microtubules are progressively broken down and cells lose their ability to follow the chemotactic gradient. The relationship between STHMN1 and p27 was identified in previous studies.^19^,^20^ Researchers reported that STHMN1 binds to p27 cells, and that cells with strong STHMN1 expression and weak p27 expression display increased proliferation and invasion capacity in tumor tissue. STMN1 was also shown to be activated by the Ras-mitogen-activated protein kinase and hedgehog signaling pathway in some studies.^21^ Researchers observed thiostrepton got over to TBR by blocking transition from G1 to S phase in cell lines.^22^ Treatment with low-dose anti-STHMN1 therapy and taxane was shown to halt tumor invasion in a breast cancer xenograft.^23^

Some studies reported that expression of the non-phosphorylated mutant leads to breakdown of the spindle assembly checkpoint and cell cycle arrest, which may be the underlying mechanism of taxane resistance.^19^,^20^,^23^

In our study, strong STHMN1 expression was identified in 26/30 (86.6%) of the patients while 4/30 (13.2%) of the cases had mild STMN1 expression; however, there was no difference in terms of response to treatment across these expression levels. Positive STHMN1 expression was observed in all of the tissue samples obtained from the patients in the present study. Hence, we considered that all tumor samples were the phosphorylated form of STHMN1; however we could not determine any relationship between STHMN1 expression and response to treatment. Eighteen (60%) of patients exhibited non response to TBR. A key role may be suggested for STHMN1 expression in the metastatic process as STHMN1 expression was observed in all of our patients, as seen in the literature.

### Limitations of the study

Firstly, we evaluated the relationship between response to TBR and the expression of STHMN1 and OPN by analyzing pathology specimens obtained from the patients at the time of diagnosis. Resistance to taxanes may be intrinsic or acquired due to long term exposure to this chemotherapeutic agent. We may have overlooked acquired resistance in the specimens in this study. There may be alterations in these specimens during the progression of metastatic process or the long term use of cytotoxic regimens. Specimens obtained by a second biopsy during the progressive period could have provided further findings. Taken together, to the best of our knowledge, heterogeneity of primary tumor specimens of cases diagnosed with mCRPC has not been observed to date. This issue may be clarified further in prospective studies.

Secondly, the present study was conducted with a limited number of specimens. If we had a sufficient number of patients for this study, perhaps it would have been possible to establish a statistically significant relationship between OS and the expression level of OPN. The present study does not support the results obtained both with in vivo studies and in vitro experiments conducted for this subject matter. Most of the previous studies were carried out with cancer cell lines. These cell lines are thought to be not fully reflecting all characteristics of human cancer cells. The present study is the first study in the literature to show that there is no relationship between OPN and STHMN1 expression and the response to TBR in mCRPC.

## CONCLUSIONS

The overexpression of STHMN1 and OPN are very important in cancer metastasis. There is no relationship between resistance to TBR and the expression level of OPN or STHMN1 according to our study. OPN and STHMN1 are not suitable markers to predict response to TBR. Furthermore, our findings suggest that the evaluation of these two proteins may be a useful predictor of poor prognosis in patients with mCRPC.

### Authors’ Contribution

**AA:** Conceived the idea, data acquisition, interpretation.

**AG:** Interpretation of pathological samples.

**SOG:** Did statistical analysis.
